# Hepatitis C Virus NS3 Inhibitors: Current and Future Perspectives

**DOI:** 10.1155/2013/467869

**Published:** 2013-10-27

**Authors:** Kazi Abdus Salam, Nobuyoshi Akimitsu

**Affiliations:** Radioisotope Center, The University of Tokyo, 2-11-16 Yayoi, Bunkyo-ku, Tokyo 113-0032, Japan

## Abstract

Currently, hepatitis C virus (HCV) infection is considered a serious health-care problem all over the world. A good number of direct-acting antivirals (DAAs) against HCV infection are in clinical progress including NS3-4A protease inhibitors, RNA-dependent RNA polymerase inhibitors, and NS5A inhibitors as well as host targeted inhibitors. Two NS3-4A protease inhibitors (telaprevir and boceprevir) have been recently approved for the treatment of hepatitis C in combination with standard of care (pegylated interferon plus ribavirin). The new therapy has significantly improved sustained virologic response (SVR); however, the adverse effects associated with this therapy are still the main concern. In addition to the emergence of viral resistance, other targets must be continually developed. One such underdeveloped target is the helicase portion of the HCV NS3 protein. This review article summarizes our current understanding of HCV treatment, particularly with those of NS3 inhibitors.

## 1. Introduction

In the mid-1970s, it was noticed that supply of blood was contaminated with an unidentified agent causing posttransfusion non-A, non-B hepatitis [1]. This unknown infectious agent struck intravenous drug users and blood transfusion recipients. The offender agent identified in 1989 was hepatitis C virus (HCV) and the first sequences of HCV were reported [2]. HCV is one of the leading agents that cause liver failure, and hepatocellular carcinoma and is the most relevant reason for liver transplantation. HCV infects about 3% of the world population; 130–200 million people are estimated to be chronically infected globally. Alarming news is that 350,000 people worldwide die from HCV-related disease every year [3]. For more than 20 years, HCV has been taking the attention of the health professionals, and now, well recognized that HCV is actually a major global health problem. Recently, health professionals determined the worldwide prevalence of HCV in comparison with HIV. The global prevalence of HCV estimates is 400,000 chronically infected subjects in Australia and Oceania, 14 million in the United States of America, 16 million in the Middle East, 17.5 million in Europe, 28 million in Africa, and 83 million in Asia [4]. Therefore, novel and effective inventions with fewer adverse effects are required for the prevention and control of HCV. The main goal of this review article is to be updated with the current treatments of HCV, putting an emphasis on the HCV NS3 protease and NS3 helicase inhibitors.

## 2. HCV Translation and Polyprotein Processing

**Figure 1:**
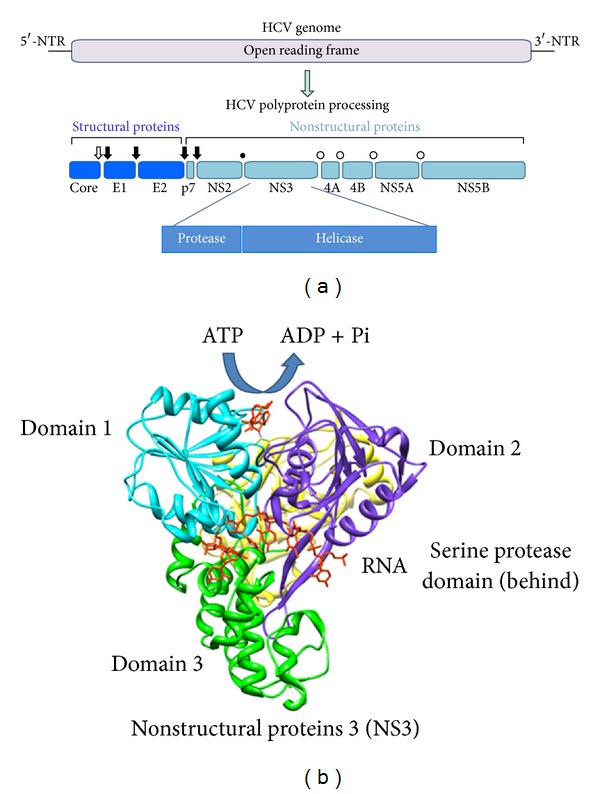
HCV genome and polyprotein processing. (a) Open arrow, closed arrows, closed circle, and open circles indicated signal peptide peptidase, signal peptidase, NS2 autoprotease, and NS3-4A serine protease cleavage site(s), respectively. (b) This figure was drawn by UCSF Chimera (http://www.cgl.ucsf.edu/chimera/), a software program for visualizing molecules, with the structural data from Protein Data Bank (PDB) ID 3O8R. Each domain of NS3 was color-coded. Both blue and purple represent helicase core domain, and green and yellow indicate C-terminal region and protease domain, respectively. ADP and RNA were drawn in red as ligands.

HCV belongs to the founding member *Hepacivirus* genus of the family *Flaviviridae* [2, 5]; it is a positive sense single-stranded RNA virus with seven genotypes and more than 90 different subtypes [6]. The viral genome is 9600 nucleotides (nt) in length, which contains a 5′-nontranslated region (NTR) with an internal ribosome entry site (IRES), 3′-NTR and encode a single polyprotein containing 3000 amino acids, and is positioned between 5′-NTR and 3′-NTR. The translation of the polyprotein is initiated by an internal ribosome entry site (IRES) present at the 5′-NTR [7]. Unlike eukaryotic mRNA, HCV genome which lacks a 5′ cap translation depends on IRES that directly binds with 40S ribosomal subunits, inducing conformational changes in the 40S subunits [8]. The IRES-40S complex then recruits eukaryotic initiation factor (eIF) 3 and the ternary complex of Met-tRNA-eIF2-GTP to form a noncanonical 48S intermediate before a kinetic slow transition to the translationally active 80S complex [9, 10]. Once the formation of initiation complex takes place, the genome of HCV is translated to produce a large polyprotein that undergoes proteolytic cleavages with specific viral and cellular proteases to form 10 individual viral proteins, each of which has specific functions in viral life cycle (Figure 1). The N-terminal one-third of the polyprotein encodes the virion structural proteins; the core protein (C) forms the viral nucleocapsid and envelopes glycoproteins E1 and E2, involved in receptor binding required for viral entry into the hepatocyte [11]. A small integral membrane protein, p7, functions as an ion channel [12, 13]. The remaining portion of the genome encodes 6 important nonstructural (NS) proteins: NS2, NS3, NS4A, NS4B, NS5A, and NS5B, which coordinate the intracellular processes of the viral life cycle. Host endoplasmic reticulum (ER) derived signal peptidase cleavages the mature structural proteins among the junctions C/E1, E1/E2, and E2/p7. Signal peptide peptidase releases core from E1 signal peptide. The p7/NS2 junction is also cleaved by signal peptidase within the NS region. Two viral enzymes, the NS2 autoprotease and the NS3-4A serine protease, are involved further in the proteolytic processing of NS proteins. The NS2 autoprotease cleaves at the NS2/3 site, whereas the NS3-4A serine protease, which requires the NS4A protein as cofactor for functioning properly, cleaves at all downstream junctions. Another small protein that encodes HCV genome is called F (frame shift) or ARFP (alternative reading frame protein), but its precise roles in viral life cycle are unknown [14]. 

## 3. The Functions of HCV NS3 Proteins

NS3 is a multifunctional protein (amino acids 1–631) with serine protease activity at the N-terminal (aa 1–180) and a nucleoside-triphosphatase- (NTPase-) dependent RNA helicase activity (NS3 NTPase/helicase) at the C-terminal (aa 181–631). Both enzyme activities have been well defined and high-resolution structures have been solved [15]. The C-terminus of NS3 encodes a DExH/D-box RNA helicase. NS3 helicase hydrolyzed NTP as an energy source to unwind double-stranded RNA in a 3′ to 5′ direction during replication of viral genomic RNA [16]. Structural analysis of NS3 revealed the unidirectional translocation and proposed a new function of NS3 as translocase, considering feasible strategies for developing specific inhibitors to block the action of NS3 helicase [17]. The activity of NS3 helicase can be regulated by interactions between the serine protease and helicase domains of NS3 [18, 19], indicating that these two enzyme activities may be somehow coordinated during replication. The function of the HCV helicase is unknown; it has been shown that without functional helicase domains, HCV cannot replicate in cells. It may be involved in the initiation of RNA synthesis on the HCV genome RNA, which contains stable 3′-terminal secondary structure in dissociation of nascent RNA strands from their template during RNA synthesis or in displacement of proteins or other *trans*-acting factors from the RNA genome. It has been now well recognized that both activities of NS3 protein are required for the replication of virus; they are considered as attractive target sites for the development of direct-acting antivirals (DAAs) therapies. NS5B is the viral RNA-dependent RNA polymerase [20], another promising anti-HCV target site. NS5A is a phosphoprotein specifically [21] capable of interacting with the 3′-NTR of the HCV genome [22], other nonstructural proteins [23], and numerous cellular proteins [24, 25]. NS5A also functions in virus assembly [26, 27]. NS4B is an integral membrane protein that is required for the assembly of the “membranous web,” the organelle used for RNA replication [28, 29]. NS4A is a cofactor for NS3 that directs the localization of NS3 and modulates its enzymatic activities [30]. 

## 4. Current Treatment for HCV Infection

A combination of pegylated interferon and ribavirin is still the only choice for the treatment of hepatitis C. Depending on the genotypes, this standard of care (SOC) increased the sustained virologic response (SVR) and defined the HCV RNA levels undetectable in the blood 24 weeks posttreatment, from ~5% to ~40–80%. In HCV genotype 1 infected patients, those with high viral loads, mostly null responders or relapsers, the SOC treatment with pegylated interferon plus ribavirin for 48 weeks achieves 50% SVR [31–35]. On the other hand, the SOC treatment with pegylated interferon plus ribavirin for 24 weeks up to 80% achieves SVR in the HCV genotype 2 infected patients. However, current SOC is associated with severe side effects including rash, nausea, anemia, and depression. 

The preventive measures against HCV include the development of HCV vaccine which may be one good idea. This is a challenging job because HCV has a great ability to change its amino acid and evade the immune response, which is-10 fold higher than HIV [3]. The development of HCV vaccine is now in progress [36]. In 2011, the US Food and Drug Administration approved two new antivirals, boceprevir and telaprevir, which was a milestone in HCV research. They inhibit an important viral protein, the NS3-4A protease. The drugs are designed in such a way that specifically attack HCV genotype 1, which is considered one of the most prevalent genotypes, accounting for about 60% of global infections, and the least responsive to current treatment. This new standard of care, a combination of boceprevir or telaprevir with peg-IFN plus ribavirin, has been approved for elimination of HCV infection in the USA, Europe, and Japan [37–40].

Anti-HCV DAAs can be classified into several categories: (1) HCV NS3-4A serine protease inhibitors, (2) HCV NS3 NTPase/helicase inhibitors, (3) HCV NS5B polymerase inhibitors, (4) HCV NS5A inhibitors, and others.

### 4.1. HCV NS3 Protease Inhibitors

HCV NS3-4A protease inhibitors (NS3-4A PIs) are classified into two groups. (1) The first generations PIs (boceprevir and telaprevir) are the linear *α*-ketoamide derivatives. These two inhibitors formed a covalent bond with the active site of the enzyme in a reversible way. Boceprevir and telaprevir are considered the first two DAAs that come to the HCV drug market, and are approved by FDA for the treatment of HCV genotype 1 infected patients as triple therapy with conventional approaches. (2) The second generations of PIs are mostly linear and macrocyclic noncovalent inhibitors of the NS3-4A enzyme. To date, both generations of PIs are highly potent inhibitors of the NS3-4A enzyme. It is known that the advantages of the second generations of PIs over the first generations are their convenience and improved side effects profile. As the resistance mutations are crucial issues in HCV therapy, unfortunately, they share the same basic resistance mutations that are generated by the first generations of PIs. Only two exceptional drugs, MK-5272 and ACH-2684, do not share the same resistance mutations, are now in clinical investigations (Table 1). ABT-450/r with potent clinical effects achieved SVR through 36 weeks of posttreatment observation, raising the possibility to treat hepatitis C with interferon-free regimens in HCV genotype 1 infected patients [41]. Simeprevir (TMC435) is being under investigated macrocyclic noncovalent NS3-4A protease inhibitor that is currently in Phase III clinical development. Clinical data showed that the addition of TMC435 to the SOC significantly increased the SVR [42]. Faldaprevir (BI 201335) is an inhibitor of HCV NS3-4A protease and is undergoing Phase III clinical trials [43, 44]. The major pharmacologic properties of clinically developed NS3-4A protease inhibitors are summarized in Table 2.

Currently, many NS3 protease inhibitors with various combinations of NS5A and polymerase inhibitors, with or without ribavirin, are being clinically investigated. For example, a study of the protease inhibitor asunaprevir in combination with the NS5A inhibitor daclatasvir, administered for genotype 1a or 1b infected patients, showed the eradication of the virus in 4 out of 11 patients (36%) [45]. Another report, which used the same regimen but only in patients with genotype 1b infection, achieved SVR 90% [46]. These two studies clearly demonstrated the effects of HCV subtype on the response to a regimen that consists entirely of direct-acting antiviral agents. Therefore, it may be feasible to treat HCV without interferon or ribavirin. 

### 4.2. HCV NS3 NTPase/Helicase Inhibitors

The structure of the NS3 helicase is also available and well characterized. However, the developments of NS3 helicase inhibitors have been slow. This target is traditionally difficult as evidenced by the fact that no helicase inhibitors have been approved for clinical use. The main issue might be toxicity because the motor domains of HCV helicase are conserved to that of cellular proteins. As a result, more attention should be given to find inhibitors that bind sites rather than the conserved regions of cellular enzymes without affecting cellular ATPases or GTPases. Recently, a good number of high-throughput screening systems (HTS) have been developed to screen potential inhibitors that specifically inhibit essential activities of NS3. Many world renowned laboratories are engaged to study the helicase portion of NS3 as a possible HCV drug target over the last 17 years. Several studies have revealed that NS3 is essential for viral replication, both in whole animal and replicon model [47, 48]. Mutations in HCV RNA are unable to replicate in subgenomic replicons, which further validates the necessities of NS3 helicase in viral life cycle. NS3 helicase has unique property that plays a more complex role in viral replication. NS3 helicase unwinds both double-stranded DNA and duplex RNA, but typically most helicases do not unwind both. It is known that there is no DNA stage in HCV replication and replication occurs outside the nucleus; the biological importance of the NS3 helicase's ability to unwind DNA remains elucidate. 

The ATP and RNA binding sites are the most promising targets on HCV. To the best of our knowledge, very limited numbers of small molecules have been reported in the literature over the past years and fewer structure-activity relationships data are available. Because NS3 helicase seems to key cellular motor proteins, monitoring ATP hydrolysis is the early screening assays to screen potential inhibitors that yielded few specific hits. However, recent screens of small chemical libraries through HTS have identified some valuable compounds that inhibit HCV catalyzed DNA unwinding, NTPase-dependent RNA helicase, and RNA binding ability, some of which also prevent HCV replicon in cells. Major NS3 helicase inhibitors with their helicase inhibitory activity employing both DNA or RNA substrate and ATPase activities are discussed in Table 3.

Halogenated benzimidazoles and benzotriazoles such as dichloro(ribofuranosyl) benzotriazole (DRBT) and tetrabromobenzotriazole (TBBT) both inhibit HCV helicase catalyzed DNA unwinding with IC_50_ of 1.5 and 20 *μ*M, respectively. When employing RNA substrate, only TBBT inhibits RNA unwinding with IC_50_ of 60 *μ*M [49]. In another report, the efficacies of TBBT and DRBT were tested in four different HCV genotype 1b replicon systems. Depending on the cell line, TBBT inhibits HCV replicons with IC_50_ ranging from 40 to 65 *μ*M and DRBT inhibits HCV replicons with IC_50_ ranging from 10 to 53 *μ*M [50]. 

Soluble blue HT inhibits NS3 catalyzed DNA unwinding with an IC_50_ of 40 *μ*M [51]. After several rounds of structural refinement, discovered one of the soluble blue HT derivatives, compound **12**, which is a good anti-HCV agent with an IC_50_ of 10.1 *μ*M and EC_50_ value of 2.72 *μ*M against HCV NS3 catalyzed DNA unwinding and replicon Ava.5/Huh-7 cells, respectively [51].

Ring-expanded “fat” nucleosides (RENs) inhibit HCV and related *Flavivirus* helicase, including the West Nile virus (WNV) and Japanese encephalitis virus (JEV). They catalyzed HCV DNA unwinding with IC_50_ in the 7–11 *μ*M range and HCV helicase catalyzed RNA unwinding with IC_50_ of 5.5–12 *μ*M. In this paper, RENs demonstrated different selectivity profiles between the viral enzymes [52]. 

Another nucleoside, the compound **4** (4-carbamoyl-5-[4,6-diamino-2,5-dihydro-1,3,5-triazin-2-yl]imidazole-1-*β*-D-ribofuranoside), inhibits helicase catalyzed DNA unwinding against WNV and HCV with IC_50_ of 23 and 37 *μ*M, respectively, but it had no effect on helicase catalyzed RNA unwinding. It was a surprise that no activity was observed against the NTPase/helicase of either DENV or JEV irrespective of whether RNA or a DNA substrate was employed [53].

QU663 inhibits HCV helicase catalyzed DNA unwinding with a *K*_*i*_ of 750 nM, competing with the nucleic acid substrate without affecting ATPase function, even at high concentrations. Docking studies showed that by interacting with the putative binding site QU663 induced a similar conformational shift [54]. 

Small peptide inhibitor, 14 amino acid-long peptide (p14), revealed a basic amino acid stretch corresponding to motif VI of HCV, WNV, and JEV of NTPase/helicase. This peptide inhibited the HCV unwinding activity of the enzyme with an IC_50_ of 0.2 *μ*M employing DNA substrate. The order of inhibitory effects was HCV > WNV > JEV. The binding of the peptides does not interfere with the NTPase activity of the enzymes [55].

Tropolone derivatives have been screened as inhibitors of HCV helicase catalyzed DNA unwinding. The derivative of tropolone, called 3,7-dibromo-5 morpholinomethyltropolone (DBMTr), acts with an IC_50_ of 17.6 *μ*M. It has no effect on HCV helicase catalyzed ATP hydrolysis [56] nor HCV helicase catalyzed RNA unwinding [57]. The authors also mentioned that DBMTr might be developed as potent inhibitor of the HCV helicase due to its low toxicity to yeast cells [56]. 

Acridone derivatives have also been screened as inhibitors of HCV helicase catalyzed DNA unwinding with IC_50_ between 1.5 and 20 *μ*M. These compounds also inhibit replication of HCV (EC_50_ 1–10 *μ*M) and are not particularly toxic to cells [58, 59]. 

The thiazolpiperazinyl derivative compound **23** inhibits the helicase activity with an IC_50_ of 110 *μ*M, using DNA substrate. None of the compounds were able to inhibit the NS3 NTPase activity. Testing in the subgenomic HCV replication, it exhibited EC_50_ of 3 *μ*g/*μ*L and CC_50_ > 50 *μ*g/*μ*L [60]. 

1-N,4-N-bis[4-(1H-Benzimidazol-2-yl)phenyl]benzene-1,4-dicarboxamide, designed as (BIP)_2_B, is a potent and selective inhibitor of HCV NS3 helicase, which inhibits unwinding reaction regardless of DNA or RNA substrate, but not ATP hydrolysis without RNA or at saturated level of RNA. (BIP)_2_B inhibited NS3 helicase from HCV genotypes 1a, 1b, 2a, and 3a. Evidence presented here shows that it directly and specifically binds to NS3 protein [61]. 

Other new tropolone derivatives, compounds **2**, **6**, and **7**, inhibit HCV catalyzed DNA unwinding (IC_50_ = 3.4–17.8 *μ*M). They are also effective in RNA replication (EC_50_ = 32.0–46.9 *μ*M) and exhibit the lowest cytotoxicity. The derivatives **2** and **7** have been shown to be resistant mutants. The effects of the compound **2** plus IFN-*γ* and compound **2** plus ribavirin combinations were evaluated in cell culture, indicating that both combinations result in an additive effect with a very slight tendency to synergy [62]. The tetrahydroacridinyl derivative **3a** is the most potent inhibitor reported to date (*K*_*i*_ = 20 nM). It did not show inhibition towards the ATPase activity of NS3 up to 100 *μ*M [63]. 

Manoalide was originally identified as an inhibitor of phospholipase A_2_, but later it was reported that it inhibits HCV NS3 helicase activity with RNA substrate (IC_50_ = 15 *μ*M). In addition, it inhibits the NS3 ATPase and RNA binding to NS3. A direct interaction between manoalide and NS3 was presented to explain the inhibition of NS3 activities through the structural change upon its binding [64]. 

The commercially available dye thioflavine S is identified as the most potent inhibitor of NS3 catalyzed DNA and RNA unwinding. After separating into their active components, P4 inhibits unwinding, subgenomic replication with IC_50_ of 2 and 10 *μ*M, respectively, and was not toxic [65]. 

SG1-23-1, isolated from ethyl acetate extract from marine feather star, *Alloeocomatella polycladia*, exhibits the strongest inhibition of NS3 helicase activity using RNA substrate (IC_50_ = 11.7 *μ*g/mL). Interestingly, the extract inhibits interaction between NS3 and RNA but not ATPase of NS3. Moreover, it also inhibits the RNA replication with EC_50_ of 23 to 44 *μ*g/mL [66]. 

Four LOPACs Sigma's library of pharmacologically active compounds (ATA, AG 538, NF 023, and Suramin) were identified. All but AG 538 have the ability to unwind DNA (IC_50_ = 0.6–3.7 *μ*M) and RNA (IC_50_ = 0.8–8.9 *μ*M). All but NF 023 inhibited replication of subgenomic HCV replicons (EC_50_ = 18–98 *μ*M). Unfortunately, none of these inhibitors were specific to NS3 helicase [67]. 

Recently, it has been reported that an ethyl acetate extract from marine sponge *Amphimedon *sp., called C-29EA, inhibits both protease (IC_50_ = 10.9 *μ*g/mL) and helicase (IC_50_ = 18.9 *μ*g/mL) activities of HCV, but not ATPase activity. Importantly, it has been shown that the highest inhibition on viral replication is derived from genotypes 1b and 2a with EC_50_ values of 1.5 and 24.9 *μ*g/mL, respectively [68]. 

Psammaplin A (PsA) has antibacterial and antitumor activity and also inhibits a wide range of enzymes reported to date. PsA has the ability to inhibit HCV helicase catalyzed RNA unwinding (IC_50_ = 17 *μ*M) in addition to ATPase and RNA binding activity. PsA inhibited the subgenomic viral replication derived from genotype 1b and genotype 2a, with EC_50_ 6.1 and 6.3 *μ*M, respectively [69]. 

Cholesterol sulfate might be a potential inhibitor of HCV NS3 helicase, with IC_50_ of 1.7 *μ*M using RNA substrate. However, it exerted no ATPase and serine protease activity. A structure-activity study revealed that anion binding and hydrophobic region in NS3 may be targets of cholesterol sulfate [70]. 

 Despite the great efforts, no potent and selective NS3 helicase inhibitors have been entered for clinical use. However, some good candidates, for example, soluble blue HT derivative, compound **12** [51], QU663 [54], and acridone derivatives [58, 59] have been identified to be suitable for further development as NS3 helicase inhibitors. It is not a surprise to imagine that NS3 helicase inhibitors will dominate HCV research in the near future.

## 5. Conclusions and Future Remarks

The direct-acting antiviral agents (DAAs), particularly NS3 protease inhibitors, telaprevir and boceprevir, which were approved in combination with current SOC (peg-IFN and ribavirin) for the treatment of HCV infection that significantly increased SVR, have opened a new window in HCV therapy. However, the side effects associated with this new therapy are a questionable maker. Anemia is the most frequent adverse effects with either telaprevir or boceprevir. They also exhibit strong inhibitory effect against an important drug metabolism enzyme, cytochrome P4503A4 (CYP3A4) resulting in the development of drug-drug interactions. In addition to drug resistance, the efficacies of these inhibitors differ significantly between HCV genotypes. It is well known that IFN itself has significant side effects. Another important issue arises with their short half-life and frequent dosing. With the advent of different small classes of DAAs, the future aim is to introduce an IFN-free regimen, oral cocktails of DAAs. The proof-of-concept studies presented some promising data confirming that the achievements of SVR without introducing IFN may be feasible. Thus, the combination of host and viral targeted inhibitors could be an attractive strategy in maximizing antiviral efficacy.

## Figures and Tables

**Table 1:** Current HCV NS3-4A protease inhibitors/drugs in pipeline (/r means boosted by ritonavir).

Mechanism	Inhibitor name	Genotypic coverage	Daily dosing	Company	Status
Reversible covalent inhibitor	Incivek (telaprevir, VX-950)	1	Three times	Vertex	Approved
	Victrelis (boceprevir, SCH503034)	1	Three times	Merck	Approved

Noncovalent inhibitor	ABT-450/r	1	Once	Abbott	Phase III
	Simeprevir (TMC435)	1, 2, 5, and 6	Once	Janssen	Phase III
	Faldaprevir (BI201335)	1	Once	Boehringer Ingelheim	Phase III
	Danoprevir (RG7227)	1	Twice	Genentech	Phase II
	Vaniprevir (MK-7009)	1	Twice	Merck	Phase II
	MK-5172	1, 2	Once	Merck	Phase II
	Asunaprevir (BMS-650032)	1, 4	Once	Bristol-Myers Squibb	Phase II
	ACH-1625	1	Once	Achillion	Phase II
	GS-9256	1	Twice	Gilead	Phase II
	ACH-2684	1, 3	Once	Achillion	Phase II
	GS-9451	1a, 1b	Once	Gilead	Phase II
	Narlaprevir/r	1	Once	Merck	Phase II
	IDX320	1, 1b, 3a, and 4a	Once	Idenix	Phase II

**Table 2:** Pharmacologic properties of direct-acting anti-HCV agents in clinical development, modified by Liang and Ghany [71].

Property	NS3-4A protease inhibitors
Efficacy	High
Genotypic coverage	Narrow (second generation drugs have broader coverage)
Probability of drug resistance	High
Side effects	Substantial
Drug-drug interactions	Substantial

**Table 3:** Inhibitory effects of some NS3 helicase inhibitors.

NS3 helicase inhibitor	IC_50_ (*μ*M)			References
	Helicase			
	DNA	RNA	ATPase	
DRBT	1.5	>500	No inhibition	[49]
TBBT	20	60	No inhibition	[49]
Soluble blue HT	40	Inhibition	23.8	[51]
Ring-expanded (fat) nucleoside analogues	7–11	5.5–12	Activation	[52]
AICAR analogue (compound 4)	37	No inhibition	ND	[53]
QU663	*K*_*i*_, 0.75	ND	No inhibition	[54]
p14	0.2	ND	No inhibition	[55]
DBMTr	17.6	No inhibition	No inhibition	[56, 57]
Acridone derivatives	1.5–20	ND	No inhibition	[58, 59]
Thiazolpiperazinyl derivative (compound 23)	110	ND	>1000	[60]
(BIP)_2_B	5.4	0.7	Inhibition (in the presence of RNA)	[61]
Tropolone derivatives	3.4–17.8	ND	ND	[62]
Tetrahydroacridine derivative, 3a	*K*_*i*_, 0.02	ND	ND	[63]
Manoalide	ND	15	70	[64]
Thioflavin S	10	12	ND	[65]
SG1-23-1	ND	11.7 *μ*g/mL	No inhibition	[66]
LOPAC compounds	0.6–3.7	0.8–8.9	ND	[67]
C-29EA	ND	18.9 *μ*g/mL	No inhibition	[68]
Psammaplin A	ND	17	32	[69]
Cholesterol sulfate	ND	1.7	No inhibition	[70]

ND: not determined.
